# A Primary Pulmonary Glomus Tumor: A Case Report and Review of the Literature

**DOI:** 10.1155/2012/782304

**Published:** 2012-09-19

**Authors:** Yasushi Ariizumi, Hirotaka Koizumi, Masahiro Hoshikawa, Takuo Shinmyo, Kouji Ando, Atsushi Mochizuki, Ayako Tateishi, Masatomo Doi, Mieko Funatsu, Ichirou Maeda, Masayuki Takagi

**Affiliations:** ^1^Department of Diagnostic Pathology, St. Marianna University School of Medicine, 2-16-1 Sugao, Miyamae, Kawasaki 216-8511, Japan; ^2^Department of Respiratory Surgery, St. Marianna University School of Medicine, 2-16-1 Sugao, Miyamae, Kawasaki 216-8511, Japan

## Abstract

A case of a glomus tumor originating from the lung is reported. A 43-year-old female had undergone resection of a right lung tumor following a clinical diagnosis of carcinoid, sclerosing hemangioma, or other sarcoma. Histologically, the tumor comprised uniform small round to oval cells with centrally located nucleus, a clear cytoplasm, and apparent cell borders. The tumor also showed a focally hemangiopericytomatous pattern with irregularly branching or dilated vessels. Electron microscopy revealed smooth muscle differentiation of the tumor cells. Immunostaining further revealed that the tumor cells expressed smooth muscle actin, h-caldesmon, muscle specific actin (HHF-35), but not cytokeratin, epithelial membrane antigen, synaptophysin, or chromogranin A. Based on these findings, a diagnosis of primary pulmonary glomus tumor was established. Glomus tumors of the lung are very rare and only 21 cases have been reported to date. The histological features of the present tumor and the relevant literature are discussed.

## 1. Introduction

Glomus tumors are benign neoplasms derived from the glomus cells surrounding an arteriovenous anastomosis. These anastomoses are most frequently found in the deep dermis of the extremities, such as the subungual regions of the finger tips, palms, wrists, and toes. Glomus tumors accounts for 1.6% of all soft tissue lesions recorded at the Mayo Clinic [1]. The average age of the affected patients at presentation ranges from 20 to 40 years, although these tumors can occur at any age [2]. Although the subungual regions are the most common sites at which glomus tumors arise, they occasionally develop in the organs where glomus bodies are sparse or unrecognized, such as the gastrointestinal tract, lung, bone, adrenal gland, central nervous system, mediastinum, uterus, and vagina [3]. We here describe a rare case of a pulmonary glomus tumor, including its pathological features, and review the current literature on these cancers.

## 2. Case Report

**Figure 1:**
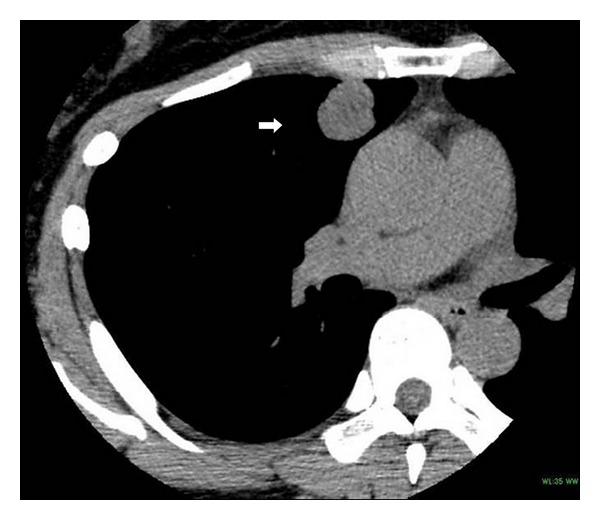
CT of the chest revealing a 2.0 × 2.0 cm nodular mass on the right peripheral upper lobe.

A 43-year-old female was admitted to our hospital with an abnormal lung shadow in the right upper field that had been detected three months previously. She was a nonsmoker and had no history of respiratory diseases. Laboratory data and tumor markers were within normal ranges. A chest computed tomography (CT) scan detected a nodular lesion of about 2.0 cm in diameter in the right upper lobe (Figure 1). Preoperative diagnoses included carcinoid, sclerosing hemangioma, and sarcomas not otherwise specified. Thoracoscopic removal of the lung with a frozen section (intraoperative rapid diagnosis) was performed. The tumor comprised blood vessels of varying sizes surrounded by uniformly arranged small round cells with hemorrhage foci. From these findings, we made an intraoperative diagnosis of sclerosing hemangioma.

**Figure 2:**
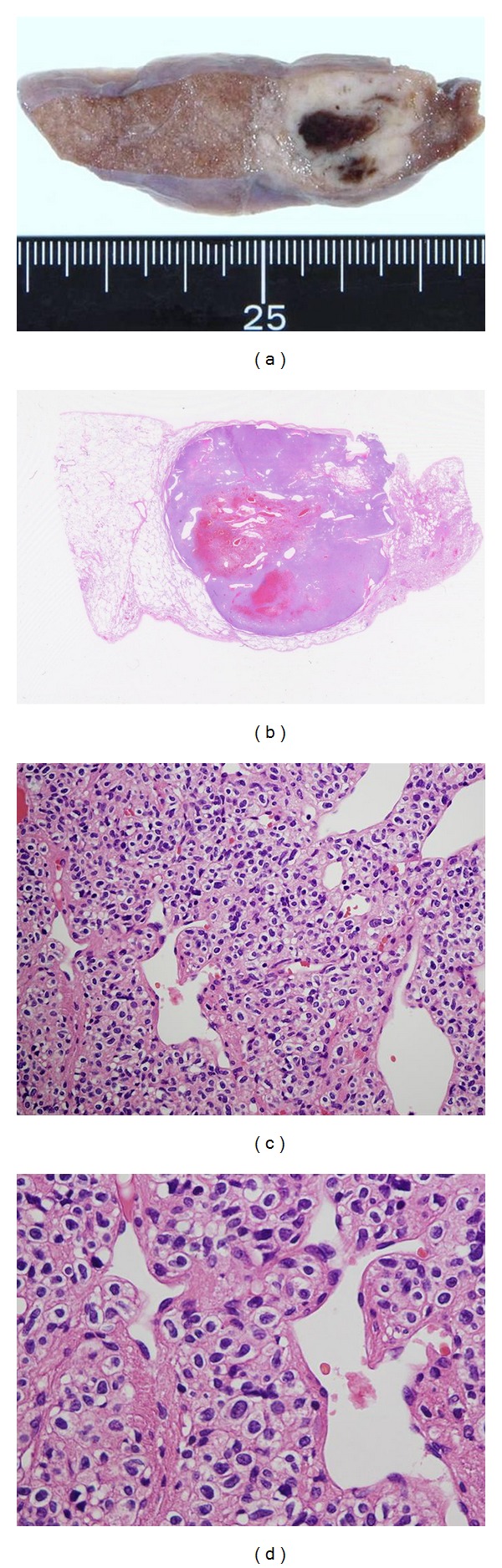
(a, b) The excised tumor, measuring 2.0 × 2.0 cm, appeared as a well-circumscribed nodular lesion with a whitish color and focal central hemorrhage. (c, d) Histological findings for the removed tumor revealed uniform small round to oval cells with a centrally located nucleolus, a clear cytoplasm, and clear cell borders. Branching vessels with a hemangiopericytomatous pattern (HPC-pattern) were also evident.

The removed tumor was solbid and whitish, measured 2.0 × 2.0 cm, and showed focal hemorrhaging and no necrotic changes (Figures 2(a) and 2(b)). Histologically, the tumor consisted of uniform small round to oval cells with centrally located nuclei, clear cytoplasms, and the appearance of cell borders. No nuclear atypia, mitotic figures, or necroses were observed. The excised tumor had irregularly branched or dilated vessels that formed the so-called hemangiopericytomatous pattern (Figures 2(c) and 2(d)). These histologic features were compatible with those of a carcinoid, sclerosing hemangioma, hemangiopericytoma, or a glomus tumor. By electron microscopy, pinocytotic vesicles and dense patches were revealed along the basement membranes (Figure 4), suggestive of smooth muscle differentiation of the tumor cells. 

**Figure 3:**
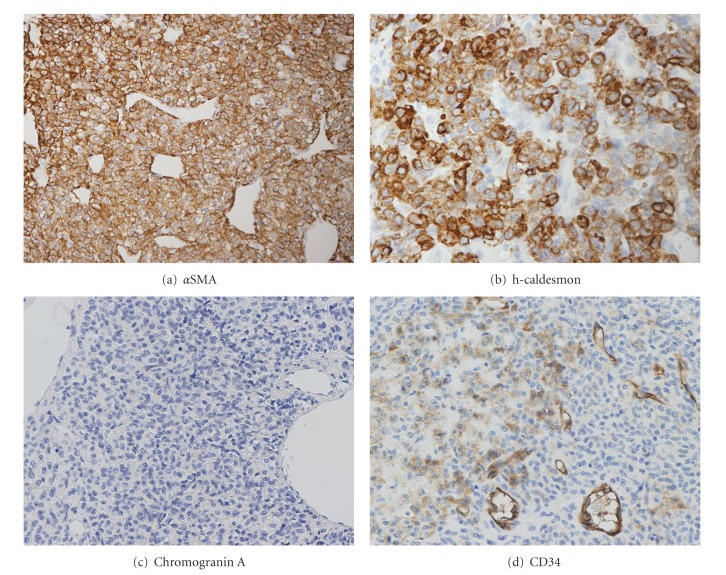
(a–c) Immunostaining revealing that the tumor cells showed a diffuse positive expression of *α*SMA and h-caldesmon at the cell membrane and cytoplasm but were negative for chromogranin A. (d) CD34 was found to be only focally positive in perivascular tumor cells.

**Figure 4:**
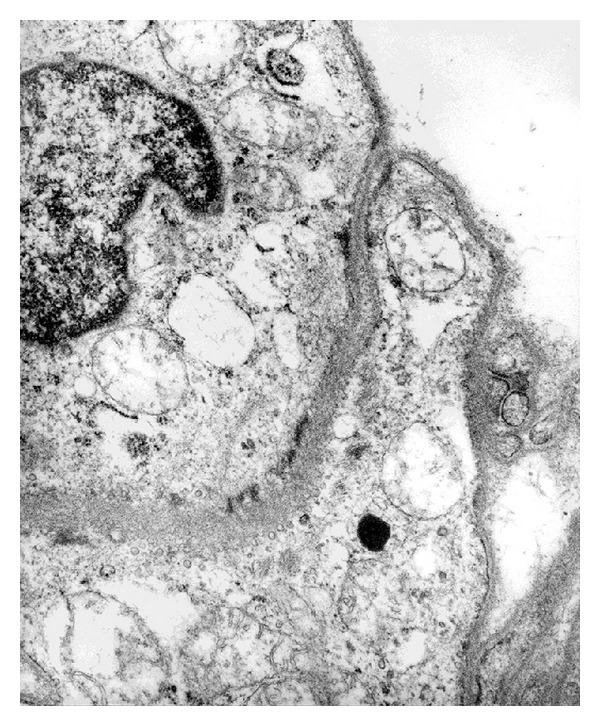
Electron microscopy revealed that the tumor cells contained pinocytotic vesicles and a dense patch lining at the basement membrane.

 By immunostaining, the tumor cells were positive for alpha smooth muscle actin, h-caldesmon, muscle specific actin (HHF-35), laminin, type IV collagen, and vimentin, but negative for cytokeratin (AE1/AE3), epithelial membrane antigen (EMA), TTF-1, surfactant apoprotein A, S-100 protein, synaptophysin, and chromogranin A (Figures 3(a)–3(d)). CD34 expression was only occasionally found in the perivascular tumor cells and the MIB-1 index was about 2%. Based on these findings, we made a final diagnosis of a glomus tumor of the lung.

## 3. Discussion

Glomus tumors are rare neoplasms that arise from the neuroarterial structure known as the glomus body [31, 32] and account for 1.6% of all soft tissue tumors [1]. The normal glomus unit is a neuromyoarterial apparatus that functions to regulate skin circulation and is found subungually in descending order on the finger tip pulp, on the base of the foot and throughout the rest of body. The most common site of glomus tumors is subungual lesion of the fingers, followed by other portions of the distal extremities including the wrist, palm, and foot. Glomus tumors have also been described in locations where the glomus body does not normally exist including the bone, chest wall, eyelid, colon, rectum, cervix, and respiratory tract [3]. We here describe a highly unusual case of a glomus tumor of the lung. To our knowledge, this is the 33th and 21th glomus tumor case to be described originating from the respiratory tract, including the trachea, bronchus, and lung, and the lung alone, respectively.  Table 1 summarizes the clinicopathological features of the 33 glomus tumors of the respiratory tract thus far reported [4–30].

Histologically, typical glomus tumors are subcategorized as solid glomus tumor, glomangioma, or glomangiomyoma, depending on the relative prominence of glomus cells, vascular structures, and smooth muscle. Glomus cells are small, uniform, and round with a centrally placed, round nucleus and an amphophilic to lightly eosinophilic cytoplasm. In soft tissues, solid glomus tumors are the most common variant, comprising approximately 75% of reported cases, followed by glomangiomas (approximately 20%) and glomangiomyomas (<5%). Malignant glomus tumors (glomangiosarcomas) are exceedingly rare and fewer than 20 soft tissue cases which have been published [2]. In the respiratory tract, the 33 glomus tumor cases reported to date consist of 26 typical and 7 malignant variants. Subcategorized diagnoses are available for 22 of the 26 typical glomus tumors, which includes 18 solid tumors (81.8%), 3 glomangiomas (13.6%), and one glomangiomyoma (4.5%, Table 1), the incidences of which are very similar to the soft tissue counterparts. The incidence of glomangiosarcomas in the respiratory tract (7/33, 21.2%) appears to be higher than that of the soft tissue counterparts, although it is uncertain whether all of these tumors fulfilled the current strict criteria for glomangiosarcomas [2]. 

A differential diagnosis of pulmonary glomus tumor must exclude carcinoid tumor, hemangiopericytoma, sclerosing hemangioma, leiomyoma, and paraganglioma. Carcinoids are often confused with glomus tumors since they have a similar morphology, although by immunostaining carcinoids, but not glomus tumors, are positive for cytokeratin and neuroendocrine markers such as chromogranin A and synaptophysin [4, 9]. Sclerosing hemangiomas are positive for TTF-1, surfactant apoprotein A, and cytokeratin. Paragangliomas are typically composed of round epithelioid cells with small nuclei and express neuroendocrine markers and S-100 protein [27]. Smooth muscle neoplasms comprise spindle cells with a fascicular pattern and express smooth muscle markers, including alpha smooth muscle actin and h-caldesmon. Hemangiopericytoma is a vascular neoplasm often misdiagnosed as a glomus tumor. These lesions show a characteristic staghorn vasculature pattern and consist of polygonal to spindle cells with elongated nuclei. Hemangiopericytomas are immunohistochemically positive for vimentin and CD34, but negative for cytokeratin and smooth muscle markers. The diffuse CD34 staining pattern found in hemangiopericytomas is very helpful in differentiating them from glomus tumors. 

In summary, we here reported a very unusual case of a primary pulmonary glomus tumor. We further find that although these neoplasms histologically resemble carcinoid tumor, hemangiopericytoma, and sclerosing hemangioma, careful morphologic observation and immunostaining for appropriate markers should enable clinicians to distinguish between these lesions. 

## Figures and Tables

**Table 1:** Clinopathological features of glomus tumor in the respiratory tract.

Author (year)	Age/sex	Symptoms	Size (cm)	Location	Biopsy diagnosis	Final diagnosis	Subclassification	Prognosis
Tang et al. (1978) [4]	67/F	Epigastralgia	6.5	Lung	N/A	Typical GT	Glomangioma	9 m FOD
Alt et al. (1983) [5]	34/M	ASX	2	Lung	HPC or GT	Typical GT	Solid GT	N/A
Koss et al. (1998) [6]	50/M	ASX	1.1	Lung	N/A	Typical GT	Solid GT	N/A
	41/M	ASX	1.5	Lung	N/A	Typical GT	Solid GT	47 m FOD
Watanabe et al. (1998) [7]	43/M	Hoarseness	2	Bronchus	Adenoma	Typical GT	Solid GT	20 m FOD
Oizumi et al. (2001) [8]	48/M	Hemosputum	0.7	Bronchus	GT	Typical GT	Solid GT	3 m FOD
Gaertner et al. (2000) [9]	20/M	Pneumo-thorax	1.4	Bronchus	Carcinoid	Typical GT	N/A	9 m FOD
	65/M	ASX	3	Lung	N/A	Typical GT	N/A	5 y FOD
	40/M	ASX	1.1	Lung	HPC suspect	Typical GT	N/A	6 m FOD
	69/M	Hemoptysis	9.5	Lung	N/A	GAS	GAS	68 w DOD
Folpe et al. (2001) [10]	38/M	ASX	3.8	Lung	N/A	GAS	GAS	N/A
	9/F	ASX	4.5	Lung	N/A	GAS	GAS	5 y AWD
Yilmaz et al. (2002) [11]	29/M	Cough	1.5	Lung	Carcinoid	Typical GT	Solid GT	17 m FOD
Hishida et al. (2003) [12]	53/M	Cough	2.5	Lung	Atypical cells	GAS	GAS	23 m FOD
Zhang and England (2003) [13]	29/M	CD	2.5	Lung	Adenoma	Typical GT	Solid GT	N/A
Ueno et al. (2004) [14]	50/M	ASX	4	Lung	N/A	Typical GT	Solid GT	N/A
de Weerdt et al. (2004) [15]	37/M	Cough	N/A	Bronchus	N/A	Typical GT	Solid GT	3 m FOD
Yu et al. (2004) [16]	47/M	Chest pain	N/A	Trachea	GT	GAS	GAS	N/A
Vailati et al. (2004) [17]	40/M	ASX	5	Bronchus	N/A	Typical GT	Solid GT	1 m FOD
Takahashi et al. (2006) [18]	67/M	Cough	3.1	Bronchus	Carcinoid	Typical GT	Solid GT	8 m FOD
Sousa and Carvalho (2006) [19]	62/M	Dyspnea	1.9	Lung	N/A	Typical GT	N/A	N/A
Katabami et al. (2006) [20]	56/M	Hemoptysis	5.5	Bronchus	Carcinoid	Typical GT	Glomangiomyoma	12 m FOD
Rössle et al. (2006) [21]	64/M	ASX	3.5	Lung	N/A	Typical GT	Glomangioma	N/A
Kapur et al. (2007) [22]	34/M	Cough	2.3	Bronchus	Carcinoid	Typical GT	Solid GT	N/A
Dalfior et al. (2008) [23]	55/M	ASX	1.1	Lung	N/A	Typical GT	Solid GT	N/A
Filice et al. (2008) [24]	69/M	Hemoptysis	2	Bronchus	Angiomatous lesion	Typical GT	Solid GT	N/A
Akata et al. (2008) [25]	39/M	Cough	2.5	Bronchus	GT	Typical GT	Solid GT	6 y FOD
De Cocker et al. (2008) [26]	21/F	ASX	2.5	Lung	N/A	Typical GT	Solid GT	N/A
Nakajima et al. (2010) [27]	30s/M	Hemosputum	1.5	Bronchus	Carcinoid	Typical GT	Solid GT	10 m FOD
Kleontas et al. (2010) [28]	74/M	Cough	3.4	Lung	GT	GAS	GAS	12 m FOD
Zhang et al. (2010) [29]	48/M	Cough	3.5	Lung	Myogenic tumor	GAS	GAS	4 d DOOD
Santambrogio et al. (2011) [30]	39/M	ASX	N/A	Lung	Neuroendocrine tumor	Typical GT	Glomangioma	51 m FOD
Present case	43/M	ASX	2	Lung	Sclerosing hemangioma	Typical GT	Solid GT	6 m FOD

ASX: asymptomatic, CD: chest discomfort, N/A: not available, HPC: hemangiopericytoma, GT: glomus tumor, GAS: glomangiosarcoma, FOD: free of disease, DOD: dead of disease, AWD: alive with disease, DOOD: dead on other disease.
